# Supplementary material for: Association between vision impairment and mortality: protocol for a systematic review and meta-analysis
Source: BMJ Open. 2020 Jun 21;10(6):e037556. doi: 10.1136/bmjopen-2020-037556 (PMC7311038; doi:10.1136/bmjopen-2020-037556)
Supplement: Author's manuscript [file bmjopen-2020-037556.draft_revisions.pdf]

BMJ Open is committed to open peer review. As part of this commitment we make the peer review history of every article we publish publicly available.

When an article is published we post the peer reviewers' comments and the authors' responses online. We also post the versions of the paper that were used during peer review. These are the versions that the peer review comments apply to.

The versions of the paper that follow are the versions that were submitted during the peer review process. They are not the versions of record or the final published versions. They should not be cited or distributed as the published version of this manuscript.

BMJ Open is an open access journal and the full, final, typeset and author-corrected version of record of the manuscript is available on our site with no access controls, subscription charges or pay-per-view fees (http://bmjopen.bmj.com).

If you have any questions on BMJ Open's open peer review process please email info.bmjopen@bmj.com

BMJ Open

The Association Between Vision Impairment and Mortality: Protocol for a Systematic Review and Meta-Analysis

Journal:	*BMJ Open*
Manuscript ID	bmjopen-2020-037556
Article Type:	Protocol
Date Submitted by the Author:	06-Feb-2020

Complete List of Authors:	Ehrlich, Joshua; University of Michigan, Ophthalmology and Visual Sciences; University of Michigan, Institute for Healthcare Policy and Innovation<br>Ramke, Jacqueline; The University of Auckland, School of Optometry and Vision Science; London School of Hygiene and Tropical Medicine, International Centre for Eye Health<br>Macleod, David; London School of Hygiene and Tropical Medicine, International Centre for Eye Health; London School of Hygiene and Tropical Medicine, MRC Tropical Epidemiology Group<br>Swenor, B; Johns Hopkins University, Ophthalmology; Johns Hopkins, Epidemiology<br>Burn, Helen; Stoke Mandeville Hospital<br>Lee, Chan; London School of Hygiene and Tropical Medicine, International Centre for Eye Health; Royal Liverpool University Hospital, St. Paul's Eye Unit<br>Waldock, William; University of Cambridge School of Clinical Medicine<br>Zhang, Justine; London School of Hygiene and Tropical Medicine, International Centre for Eye Health; Manchester Royal Eye Hospital<br>Gordon, Iris; London School of Hygiene and Tropical Medicine, International Centre for Eye Health<br>Congdon, Nathan; Queen's University Belfast; Sun Yat-Sen University Zhongshan Ophthalmic Center<br>Burton, Matthew J; London School of Hygiene and Tropical Medicine, International Centre for Eye Health; Moorfields Eye Hospital<br>Evans, Jennifer; London School of Hygiene and Tropical Medicine, International Centre for Eye Health

Keywords:	OPHTHALMOLOGY, EPIDEMIOLOGY, PUBLIC HEALTH

SCHOLARONE™
Manuscripts

I, the Submitting Author has the right to grant and does grant on behalf of all authors of the Work (as defined in the below author licence), an exclusive licence and/or a non-exclusive licence for contributions from authors who are: i) UK Crown employees; ii) where BMJ has agreed a CC-BY licence shall apply, and/or iii) in accordance with the terms applicable for US Federal Government officers or employees acting as part of their official duties; on a worldwide, perpetual, irrevocable, royalty-free basis to BMJ Publishing Group Ltd ("BMJ") its licensees and where the relevant Journal is co-owned by BMJ to the co-owners of the Journal, to publish the Work in this journal and any other BMJ products and to exploit all rights, as set out in our licence.

The Submitting Author accepts and understands that any supply made under these terms is made by BMJ to the Submitting Author unless you are acting as an employee on behalf of your employer or a postgraduate student of an affiliated institution which is paying any applicable article publishing charge ("APC") for Open Access articles. Where the Submitting Author wishes to make the Work available on an Open Access basis (and intends to pay the relevant APC), the terms of reuse of such Open Access shall be governed by a Creative Commons licence – details of these licences and which Creative Commons licence will apply to this Work are set out in our licence referred to above.

Other than as permitted in any relevant BMJ Author's Self Archiving Policies, I confirm this Work has not been accepted for publication elsewhere, is not being considered for publication elsewhere and does not duplicate material already published. I confirm all authors consent to publication of this Work and authorise the granting of this licence.

For peer review only

The Association Between Vision Impairment and Mortality: Protocol for a Systematic Review and Meta-Analysis

Authors:

Joshua R. Ehrlich[1,2], Jacqueline Ramke[3,4], David Macleod[3,5], Bonnielin K. Swenor[6,7], Helen Burn[8], Chan Ning Lee[3,9], William Waldock[10], Justine H. Zhang[3,11], Iris Gordon[3], Nathan Congdon[12,13,14], Matthew Burton[3,15], Jennifer R. Evans[3]

Affiliations:

1. Department of Ophthalmology and Visual Sciences, University of Michigan, Ann Arbor, MI, USA
2. Institute for Healthcare Policy and Innovation, University of Michigan, Ann Arbor, MI, USA
3. International Centre for Eye Health, London School of Hygiene and Tropical Medicine, London, UK
4. School of Optometry and Vision Science, University of Auckland, Auckland, New Zealand
5. MRC Tropical Epidemiology Group, London School of Hygiene and Tropical Medicine, London, UK
6. Wilmer Eye Institute, Johns Hopkins University School of Medicine, Baltimore, MD, USA
7. Department of Epidemiology, Johns Hopkins Bloomberg School of Public Health, Baltimore, MD, USA
8. Stoke Mandeville Hospital, Aylesbury, UK
9. St Paul's Eye Unit, Royal Liverpool University Hospital, Liverpool, UK
10. University of Cambridge School of Medicine, Cambridge, UK
11. Manchester Royal Eye Hospital, Manchester, UK
12. Queen's University Belfast, Belfast, UK
13. Orbis International, New York, NY, USA
14. Zhongshan Ophthalmic Center, Guangzhou China
15. Moorfields Eye Hospital, London, UK

Corresponding Author: Joshua R. Ehrlich, MD, MPH, 1000 Wall Street, Ann Arbor, MI, 48103, USA. joshre@med.umich.edu, phone: +1 734-763-3732

Keywords: ophthalmology, eye, global health, visual impairment, mortality

For peer review only - http://bmjopen.bmj.com/site/about/guidelines.xhtml

ABSTRACT

Introduction: Due to growth and aging of the world's population, the number of individuals worldwide with vision impairment and blindness is projected to increase rapidly over the coming decades. Vision impairment and blindness are an important cause of years lived with disability. However, the association of vision impairment and blindness with mortality, including the risk of bias in published studies and certainty of the evidence, has not been adequately studied in an up-to-date systematic review and meta-analysis.

Methods and Analysis: The planned systematic review and meta-analysis will adhere to the Preferred Reporting Items for Systematic Review and Meta-Analysis (PRSIMA) guidelines. Databases, including MEDLINE Ovid, Embase Ovid, and Global Health, will be searched for relevant studies. Two reviewers will then screen studies and review full texts to identify studies for inclusion. Data extraction will be performed, and for included studies the risk of bias and certainty of the evidence will be assessed using the Grades of Recommendation, Assessment, Development, and Evaluation (GRADE) approach. Results from included studies will be meta-analysed according to relevant sections of the Meta-analysis Of Observational Studies in Epidemiology (MOOSE) checklist.

Ethics and Dissemination: This review will only include published data; therefore, ethics approval will not be sought. The findings of this review and meta-analysis will be published in an open-access, peer-reviewed journal and will be included in the ongoing *Lancet Global Health* Commission on Global Eye Health.

Registration details: http://osf.io/weu96

For peer review only

For peer review only - http://bmjopen.bmj.com/site/about/guidelines.xhtml

STRENGTHS AND LIMITATIONS OF THIS STUDY

- This is an up-to-date systematic review and meta-analysis to determine the nature and extent of published literature on the association of vision impairment and blindness with mortality.
- This review will comprehensively assess published peer-reviewed English-language manuscripts, with no time period or geographical restrictions.
- This will be the first review to carry out a formal assessment of risk of bias in included studies and the certainty of the evidence on this topic using the Grades of Recommendation, Assessment, Development, and Evaluation (GRADE) approach.
- A potential limitation might be the paucity of published literature on how specific levels of vision impairment contribute to mortality.
- Another potential limitation is that the complexity of pathways between eye health and mortality is unlikely to be fully described and tested in the current literature.

For peer review only

For peer review only - http://bmjopen.bmj.com/site/about/guidelines.xhtml

INTRODUCTION

More than 250 million people globally are blind or visually impaired, and the number affected is projected to increase due to growth and aging of the world's population.[1] Poor vision is associated with an increased risk of dementia, depression, falls, and loss of independence.[1,2] Some prior studies have also reported that individuals with vision impairment (VI) have an increased risk of mortality compared to those with normal vision.[3] However, an up-to-date systematic review and meta-analysis of the published literature, including a formal assessment of risk of bias and certainty of the evidence, is needed to characterize the relationship between VI and mortality globally.

In order to guide a systematic review and meta-analysis of the association of VI with mortality, we developed a theoretical framework adapted from the World Health Organization (WHO) International Classification of Functioning (ICF).[4] Our framework illustrates the possible relationship between VI and mortality, as well as the diverse mediating and moderating factors that may contribute to this association (Figure 1). As depicted, we hypothesize that VI, operationalised as a decline in visual function, is associated with mortality through its effects on systemic health (e.g. an increased risk of chronic disease, frailty, and decreased functional status). Factors such as participation (social, physical, daily activities) both impact and are impacted by visual and systemic health (e.g. VI increases the risk of social isolation, which in turn affects overall health). Finally, individual-level traits, environmental and health system characteristics, smoking, and conditions with both ocular and systemic manifestations (e.g. diabetes, stroke) may simultaneously increase the risk of both VI and mortality.

A prior systematic review and meta-analysis published in 2016 summarised findings from 29 prospective studies that assessed the association between vision and the risk of mortality.[3] That study reported that the risk of death was 36% higher in the group with the highest level of VI compared to those without VI, and that for each 0.1 increment change in logarithm of minimum angle of resolution (logMAR) the risk of death increased by 4%. However, the study had several limitations. First, it did not assess or account for the level of bias in included studies or certainty of the evidence. Additionally, three included studies assessed VI based on billing codes, and seven used self-reported VI rather objective quantifiable measures. Self-reported visual function may reflect a distinct latent construct,[5] in which case the pooled analysis of studies that assessed visual function objectively and subjectively may bias results in an unpredictable fashion. The highest level of VI was used as a predictor of mortality even though VI categories varied from study to study. Search terms also did not include specific eye conditions (e.g. glaucoma or cataract), so studies of the association between mortality and VI due to these conditions may have been omitted. Finally, several prospective studies have been published in recent years that report the association between VI and mortality in geographic regions, such as sub-Saharan Africa, that were under-represented in the prior systematic review and meta-analysis.

The study described in this protocol will seek to provide an updated review of the literature and estimate of the effect of VI on the risk of mortality. By including an assessment of the risk of bias to inform an overall judgement of the

For peer review only - http://bmjopen.bmj.com/site/about/guidelines.xhtml

certainty of the evidence, and by considering newly published studies from under-represented geographic regions, this systematic review and meta-analysis will make an important contribution to global eye health.

Objectives / Review questions

This systematic review and meta-analysis will aim to answer the following questions:

1. What is the extent, strength, and quality of the published evidence that VI is associated with the risk of all-cause mortality?
2. To what degree does VI affect the risk of all-cause mortality, and does this risk vary based on level of visual function?

METHODS AND ANALYSIS

Protocol and registration

We have drawn upon the PROGnosis RESearch Strategy (PROGRESS) framework for prognosis research in developing this protocol.[6] The protocol has been registered prospectively with the Open Science Framework (OSF) registry and can be viewed at: http://osf.io/weu96. Any future amendments to the protocol will be noted in the OSF registration. Results of the systematic review and meta-analysis described herein will be reported according to the relevant section of the Preferred Reporting Items for Systematic Review and Meta-Analysis (PRISMA) checklist for systematic reviews.[7] The completed Preferred Reporting Items for Systematic Review and Meta-Analysis Protocols (PRISMA-P) checklist is presented in Appendix 1.[8]

Criteria for considering studies to review

Types of studies: We will include published prospective and retrospective cohort studies with a baseline assessment of the exposure (vision) and longitudinal assessment of the outcome (all-cause mortality) over a period of at least 1 year. Since age is a strong risk factor for mortality and VI, estimates of the effect of VI on mortality risk must be age-adjusted. Interventional studies and studies where all participants had a specific systemic disease (e.g. diabetes) will be excluded due to the difficulty of separating the possible effect of VI on mortality from the effect of an intervention or systemic disease on mortality. Only peer-reviewed articles published in English will be included. We will not include grey literature or conference abstracts. We will consider publications from all years and geographical regions.

Types of participants*:* Men and women aged 40 years and above at the time of enrolment will be eligible for inclusion. We are restricting the population to this age-group of because of the low rate of mortality in younger individuals.

Types of prognostic factors:

The prognostic factor in this study is visual function. Visual function must have been measured using a standard objective ophthalmic clinical or research instrument, including, but not limited to visual acuity, visual fields, contrast sensitivity, and stereoacuity. If a study contains data on the effect of multiple measures of visual function (e.g. visual

For peer review only - http://bmjopen.bmj.com/site/about/guidelines.xhtml

acuity and visual fields), we will report each of these and they will be included in the meta-analysis. Studies where visual function was self-reported or determined based on billing codes will not be included.

We will consider several cut-points:

1. For visual acuity, we will consider presenting binocular visual acuity or visual acuity in the better-seeing eye (if both are reported we will use the binocular measurement). Definitions of VI will be based on the categories of VI in the WHO International Classification of Diseases.[9] People with any of the following categories of VI will be compared to those with better vision.
    a. Mild VI or worse will be defined as visual acuity < 6/12.
    b. Moderate VI or worse will be defined as visual acuity <6/18.
    c. Severe VI or worse will be defined as visual acuity <6/60.
    d. Blindness will be defined as visual acuity <3/60.
2. For other measures of vision, we will adopt study-specific definitions of VI since standardised definitions do not exist or are not widely used.

We will use the definitions of VI and blindness reported in the included studies that most closely correspond to these definitions. However, we anticipate heterogeneity in these measures across studies. For example, studies may vary in whether they consider uncorrected, presenting, or best-corrected visual acuity; they may include measures of visual fields, stereoacuity, and/or contrast sensitivity in their definition; they may consider the worse-seeing eye, better-seeing eye, or binocular visual acuity; and they may employ different categorical definitions of VI and blindness. We will be inclusive but will explore this heterogeneity in meta-regression analyses (see below). When available, we will also use continuous measures of vision (e.g. logMAR or log contrast sensitivity) in our analyses.

For peer review only

Types of outcome measures:

Outcome

1. The outcome is all-cause mortality one or more years after baseline assessment of vision. Mortality may be reported using different measures of effect size and we will include all measures.

Ascertainment of death may be made by any method, including but not limited to review of vital records (e.g. death certificates) or report by an informant. We have chosen to include studies that ascertained death by informant report since not all countries provide access to complete vital records and we seek to include studies from all regions of the world.

Measures of effect

To determine the association between VI and mortality, we will extract age-adjusted measures of effect reported in each included study. All measures of effect must be age-adjusted. When available, we will extract measures that have adjusted for other theoretical confounders (e.g. socioeconomic status, smoking status) as depicted in our conceptual

For peer review only - http://bmjopen.bmj.com/site/about/guidelines.xhtml

framework (Figure). To the extent that it is possible to do so, we will choose measures of effect that are not adjusted for likely mediators on the pathway between VI and mortality, such as overall health or functional status.

Search method for identification of studies

Electronic searches: We will search the following electronic databases:

1. MEDLINE Ovid (1946 to present);
2. Embase Ovid (1980 to present); and
3. Global Health (1973 to present).

The full electronic search strategy for MEDLINE Ovid is included in Appendix 2. We will not limit the search by date. As noted above, the search will be limited to English language articles and will not include conference abstracts or grey literature.

Searching other resources: We will identify additional studies by searching the reference lists of relevant publications identified through the electronic searches and by searching any prior review articles on this topic.

For peer review only

Data collection and analysis

Selection of studies: Two review authors will independently screen search results based on title and abstract and will remove reports that clearly do not fall into the scope of this review. Disagreements will be resolved by discussion and consultation with another author as needed. We will acquire the full text of all publications appearing to potentially meet criteria for inclusion in this review. Two review authors will screen all of these reports for method of visual function assessment, type of study design, duration of follow-up, and ascertainment of death. Any disagreements will be discussed and if they cannot be resolved will be arbitrated by a third review author. Screening of search results will be conducted using Covidence systematic review software (Veritas Health Innovation, Melbourne, Australia; available at www.covidence.org).

Data extraction and management: Data extraction will be guided by the relevant sections of the CHecklist for critical Appraisal and data extraction for Systematic Reviews of prediction Modelling Studies (CHARMS) checklist.[10] Two review authors will independently extract the following data from each included study: study design, participant characteristics, study population and size, study setting, study dates, follow-up duration, diagnostic and ascertainment methods, study attrition, estimates of effect size, and standard errors. Any disagreements will be discussed and if they cannot be resolved will be arbitrated by a third review author. The types of data likely to be reported as estimates of effect size include hazard ratios, risk ratios, rate ratios, standardized mortality ratios, cumulative incidence rates, proportions, survival curves, and/or odds ratios. Data extraction and management will be conducted using Covidence systematic review software.

Assessment of risk of bias: Two reviewers will independently assess the risk of bias in each included study using the Quality in Prognostic Studies (QUIPS) tool.[7]. They will assess study participation, attrition, prognostic factor measurement, outcome measurement, confounding, and statistical analysis and reporting. Likely confounders include systemic health conditions that increase risk of VI and mortality (e.g. diabetes), access to medical care, socioeconomic status, and smoking status. For each QUIPS domain, we will assign a rating of low, moderate, or high risk of bias. Ratings of each of the domains in QUIPS will be considered to provide an overall risk of bias assessment for each study. Only studies receiving a rating of low risk of bias in all of the aforementioned domains will be given an overall "low" rating; any study that received one or more ratings of high risk of bias will receive an overall "high" rating; other studies will receive an overall "medium" risk of bias rating.

Measures of association: We will extract summary measures of the association between VI and risk of mortality. We anticipate that some studies will report an overall event rate for the study period, while others may provide estimates of effect size. For all estimates we will extract standard errors; If they are not reported we will extract 95% confidence intervals and use these to calculate standard errors. As noted, we will preference measures that are adjusted for theoretical confounders but not mediators of the association between VI and mortality.

For peer review only

For peer review only - http://bmjopen.bmj.com/site/about/guidelines.xhtml

Dealing with missing data: We will include studies that follow individuals with and without VI (or with varying levels of VI) for one or more years and report the proportion who died, even if there are missing data. If all of the necessary information are not found in a published study, for articles published in 2010 or later we will email the corresponding author to solicit further information. If we are unable to obtain the necessary information, we will document in the review that we attempted to contact the study authors. We will consider the sensitivity of our meta-analysis to the effect of missing data. We will analyse the data that is available rather than imputing missing data. We will document and discuss the possible effect of missing data on each study and on the overall review and meta-analysis.

Assessment of heterogeneity: Clinical heterogeneity will be assessed by comparing key participant characteristics at the study level (e.g. age, sex, ocular diagnoses). Methodological heterogeneity will also be considered, including a comparison of the risk of bias of included studies. We will assess statistical heterogeneity by inspecting forest plots and through inspection of the I^2 and Tau^2 statistics to examine the proportion of heterogeneity across studies that is due to chance. If high levels of heterogeneity are detected (I^2>50%), we will explore likely sources of this heterogeneity (see Meta-regression below). We will also assess small study effects, one of which may be publication bias, by preparing a funnel plot [11], which is a scatter plot of effect size versus precision (standard error).

Data synthesis

Data synthesis and meta-analysis approaches: Methods and results of our meta-analysis will be guided by relevant sections of the Meta-analysis Of Observational Studies in Epidemiology (MOOSE) checklist.[12] Meta-analyses will be performed using a random-effects, generic inverse variance meta-analysis model in Stata version 16 (StataCorp, College Station, TX, USA). Random effects, rather than fixed effects, models will be used since it is likely that the true effect of VI on mortality varies from study to study due to differences in study populations and contexts. The meta-analysis will be summarised using the pooled estimate, its 95% confidence interval, and between study variance (Tau^2). The meta-analysis will be performed and results will be reported for adjusted effect estimates. We will conduct meta-analyses separately for the different types of effect measures (e.g. hazard ratios, odds ratios, and risk ratios). We will assess and report the overall quality of evidence from our meta-analysis using the modified Grades of Recommendation, Assessment, Development, and Evaluation (GRADE) tool.[13]

Meta-regression: Where data permit, we will investigate the relationship between the following covariates and effect size using random-effects meta-regression:

- Sex
- average age
- method(s) used to measure visual function (visual acuity, visual field)
- duration of follow-up
- global super-region as defined in the Global Burden of Disease study [14]

For peer review only - http://bmjopen.bmj.com/site/about/guidelines.xhtml

Sensitivity analyses: We will conduct a sensitivity analysis in which studies are excluded if they received an overall high risk of bias rating or if the risk of bias could not be adequately assessed.

Patient and Public Involvement Statement

As we plan to review existing published literature only, this review will be performed without specific patient or public involvement.

ETHICS AND DISSEMINATION

Ethics approval is not required, as our review will only include published data. Findings will be published in an open-access peer-reviewed journal and a summary of results will also be included in the ongoing *Lancet Global Health* Commission on Global Eye Health.[15] We anticipate that the findings will be of considerable interest to those involved in eye health provision, as well as the general medical, public health, development, and governmental sectors.

For peer review only

REFERENCES

1 *World Report on Vision*. Geneva: World Health Organization 2019. CC BY-NC-SA 3.0 IGO

2 National Academies of Sciences, Engineering, and Medicine. *Making Eye Health a Population Health Imperative: Vision for Tomorrow*. Washington D.C.: : The National Academies Press 2016.

3 Zhang T, Jiang W, Song X, *et al.* The association between visual impairment and the risk of mortality: a meta-analysis of prospective studies. *J Epidemiol Community Health* 2016;**70**:836–42. doi:10.1136/jech-2016-207331

4 World Health Organization. *International Classification of Functioning, Disability and Health*. 1st ed. Geneva, Switzerland: : World Health Organization 2001.

5 Donoghue OA, Ryan H, Duggan E, *et al.* Relationship between fear of falling and mobility varies with visual function among older adults. *Geriatr Gerontol Int* 2014;**14**:827–36. doi:10.1111/ggi.12174

6 Hemingway H, Croft P, Perel P, *et al.* Prognosis research strategy (PROGRESS) 1: A framework for researching clinical outcomes. *BMJ* 2013;**346**. doi:10.1136/bmj.e5595

7 Moher D, Liberati A, Tetzlaff J, *et al.* Preferred reporting items for systematic reviews and meta-analyses: the PRISMA statement. *PLoS Med* 2009;**6**:e1000097. doi:10.1371/journal.pmed.1000097

8 Shamseer L, Moher D, Clarke M, *et al.* Preferred reporting items for systematic review and meta-analysis protocols (PRISMA-P) 2015: elaboration and explanation. *BMJ* 2015;**349**. doi:10.1136/bmj.g7647

9 World Health Organization. *International Classification of Diseases*. 11th rev., ICD-11. Geneva: : World Health Organization 2018.

10 Moons KGM, Groot JAH de, Bouwmeester W, *et al.* Critical Appraisal and Data Extraction for Systematic Reviews of Prediction Modelling Studies: The CHARMS Checklist. *PLOS Medicine* 2014;**11**:e1001744. doi:10.1371/journal.pmed.1001744

11 Egger M, Davey Smith G, Schneider M, *et al.* Bias in meta-analysis detected by a simple, graphical test. *BMJ* 1997;**315**:629–34. doi:10.1136/bmj.315.7109.629

12 Stroup DF, Berlin JA, Morton SC, *et al.* Meta-analysis of observational studies in epidemiology: a proposal for reporting. Meta-analysis Of Observational Studies in Epidemiology (MOOSE) group. *JAMA* 2000;**283**:2008–12. doi:10.1001/jama.283.15.2008

13 Iorio A, Spencer FA, Falavigna M, *et al.* Use of GRADE for assessment of evidence about prognosis: rating confidence in estimates of event rates in broad categories of patients. *BMJ* 2015;**350**:h870. doi:10.1136/bmj.h870

14 GBD 2017 DALYs and HALE Collaborators. Global, regional, and national disability-adjusted life-years (DALYs) for 359 diseases and injuries and healthy life expectancy (HALE) for 195 countries and territories, 1990-2017: a systematic analysis for the Global Burden of Disease Study 2017. *Lancet* 2018;**392**:1859–922. doi:10.1016/S0140-6736(18)32335-3

15 Burton MJ, Faal HB, Ramke J, *et al.* Announcing The Lancet Global Health Commission on Global Eye Health. *Lancet Glob Health* 2019;**7**:e1612–3. doi:10.1016/S2214-109X(19)30450-4

For peer review only

For peer review only - http://bmjopen.bmj.com/site/about/guidelines.xhtml

Authors' Contributions: All authors made substantive intellectual contributions to the development of this protocol. JRE, JR, MB, and JE conceptualised the review approach. JRE drafted the first version. JR, MB, and JE provided guidance to the research team. DM, BS, IG, NC, HB, CNL, WW, and JZ developed the draft further. All authors were involved in revisions of the manuscript, developing review questions, and the review design. All authors approved the final version of the manuscript. JRE and JE take overall responsibility for the content of this manuscript.

Funding Statement: MJB is supported by grants from the Wellcome Trust (207472/Z/17/Z) to MB. JR is a Commonwealth Rutherford Fellow, funded by the UK government through the Commonwealth Scholarship Commission in the UK. BKS is supported by the National Institutes of Health (K01AG052640). JRE is supported by the National Institutes of Health (K23EY027848). The Lancet Global Health Commission on Global Eye Health is supported by The Queen Elizabeth Diamond Jubilee Trust, Moorfields Eye Charity (GR001061), NIHR Moorfields Biomedical Research Centre, The Wellcome Trust, Sightsavers, The Fred Hollows Foundation, The SEVA Foundation, The British Council for the Prevention of Blindness and Christian Blind Mission. No funder had any role in the design or conduct of this work.

Acknowledgements: None.

Competing Interests: None declared.

Word Count: 2,796

For peer review only

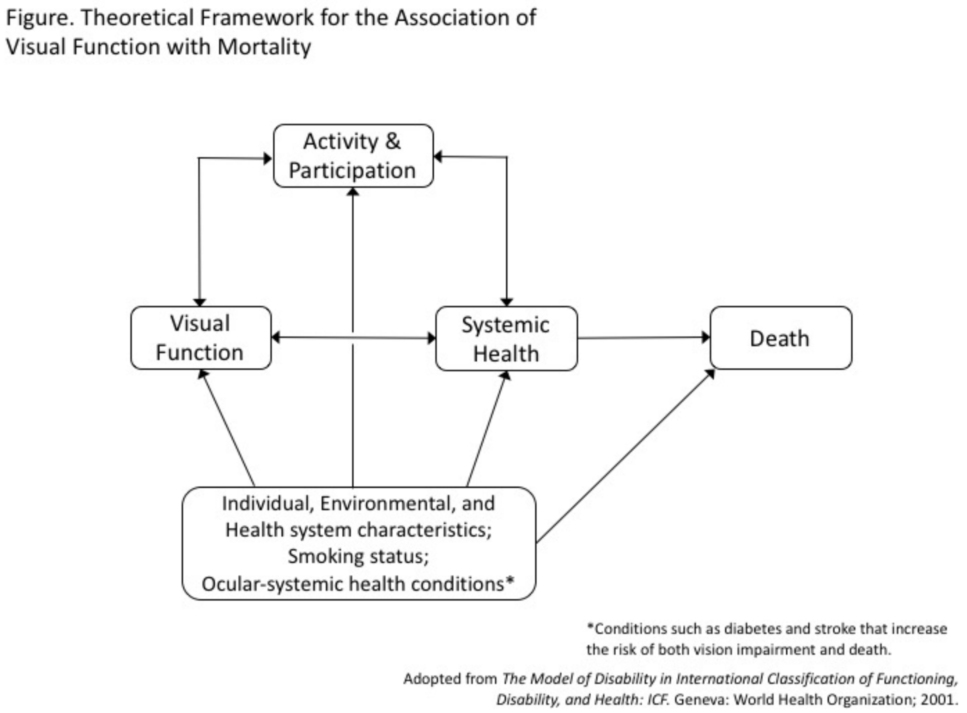

Theoretical framework depicting the hypothesized relationships between vision impairment and mortality.

254x190mm (72 x 72 DPI)

Appendix 1. PRISMA-P 2015 Checklist

Section/topic	#	Checklist item	Information reported Yes	Information reported No	Line number(s)
**ADMINISTRATIVE INFORMATION**					
**Title**					
Identification	1a	Identify the report as a protocol of a systematic review	☒	☐	1, 27, 44, 65, 97
Update	1b	If the protocol is for an update of a previous systematic review, identify as such	☒	☐	76
**Registration**	2	If registered, provide the name of the registry (e.g., PROSPERO) and registration number in the Abstract	☒	☐	39, 108
**Authors**					
Contact	3a	Provide name, institutional affiliation, and e-mail address of all protocol authors; provide physical mailing address of corresponding author	☒	☐	5-14
Contributions	3b	Describe contributions of protocol authors and identify the guarantor of the review	☒	☐	304-308
**Amendments**	4	If the protocol represents an amendment of a previously completed or published protocol, identify as such and list changes; otherwise, state plan for documenting important protocol amendments	☐	☒	N/A
**Support**					
Sources	5a	Indicate sources of financial or other support for the review	☒	☐	310-317
Sponsor	5b	Provide name for the review funder and/or sponsor	☒	☐	310-317
Role of sponsor/funder	5c	Describe roles of funder(s), sponsor(s), and/or institution(s), if any, in developing the protocol	☒	☐	316-317
**INTRODUCTION**					
**Rationale**	6	Describe the rationale for the review in the context of what is already known	☒	☐	91-94
**Objectives**	7	Provide an explicit statement of the question(s) the review will address with reference to participants, interventions, comparators, and outcomes (PICO)	☒	☐	98-101
**METHODS**					
**Eligibility criteria**	8	Specify the study characteristics (e.g., PICO, study design, setting, time frame) and report characteristics (e.g., years considered, language, publication status) to be used as criteria for eligibility for the review	☒	☐	115-121
**Information sources**	9	Describe all intended information sources (e.g., electronic databases, contact with study authors, trial registers, or other grey literature sources) with planned dates of coverage	☒	☐	170-179
**Search strategy**	10	Present draft of search strategy to be used for at least one electronic database, including planned limits, such that it could be repeated	☒	☐	Appendix 2
STUDY RECORDS					
Data management	11a	Describe the mechanism(s) that will be used to manage records and data throughout the review	☒	☐	187-189, 198-199

For peer review only

Section/topic	#	Checklist item	Information reported Yes	Information reported No	Line number(s)
Selection process	11b	State the process that will be used for selecting studies (e.g., two independent reviewers) through each phase of the review (i.e., screening, eligibility, and inclusion in meta-analysis)	☒	☐	182-189
Data collection process	11c	Describe planned method of extracting data from reports (e.g., piloting forms, done independently, in duplicate), any processes for obtaining and confirming data from investigators	☒	☐	191-199
**Data items**	12	List and define all variables for which data will be sought (e.g., PICO items, funding sources), any pre-planned data assumptions and simplifications	☒	☐	193-195
**Outcomes and prioritization**	13	List and define all outcomes for which data will be sought, including prioritization of main and additional outcomes, with rationale	☒	☐	155-160
**Risk of bias in individual studies**	14	Describe anticipated methods for assessing risk of bias of individual studies, including whether this will be done at the outcome or study level, or both; state how this information will be used in data synthesis	☒	☐	201-209
DATA					
**Synthesis**	15a	Describe criteria under which study data will be quantitatively synthesized	☒	☐	234-243
	15b	If data are appropriate for quantitative synthesis, describe planned summary measures, methods of handling data, and methods of combining data from studies, including any planned exploration of consistency (e.g., I^2, Kendall's tau)	☒	☐	211-215, 225-231, 237-241
	15c	Describe any proposed additional analyses (e.g., sensitivity or subgroup analyses, meta-regression)	☒	☐	245-254
	15d	If quantitative synthesis is not appropriate, describe the type of summary planned	☐	☒	N/A
**Meta-bias(es)**	16	Specify any planned assessment of meta-bias(es) (e.g., publication bias across studies, selective reporting within studies)	☒	☐	225-231
**Confidence in cumulative evidence**	17	Describe how the strength of the body of evidence will be assessed (e.g., GRADE)	☒	☐	241-243

For peer review only

Appendix 2: MEDLINE Ovid search strategy

1. exp eye diseases/
2. Visually Impaired Persons/
3. ((low$ or handicap$ or subnormal$ or impair$ or partial$ or disab$ or disorder$ or loss$ or limit$) adj3 (vision or visual$ or sight$)).tw.
4. ((macul$ or retina$ or choroid$) adj3 degener$).tw.
5. ((macul$ or retina$ or choroid$) adj3 neovasc$).tw.
6. (AMD or ARMD).tw.
7. ((diabet$ or proliferative or non-proliferative or pre-proliferative) adj4 retinopath$).tw.
8. (diabet$ adj2 (eye$ or vision or visual$ or sight$)).tw.
9. glaucoma$.tw.
10. cataract$.tw.
11. blindness.tw.
12. Visual Acuity/
13. visual acuit$.tw.
14. Contrast Sensitivity/
15. (contrast adj2 sensitivity).tw.
16. Depth Perception/
17. stereopsis.tw.
18. (stereo adj1 acuit$).tw.
19. Visual Fields/
20. ((visual$ or vision) adj2 function$).tw.
21. or/1-20
22. exp Mortality/
23. Death Certificates/
24. mortality.tw.
25. death$.tw.
26. (fatality or fatalities).tw.
27. or/22-26
28. Cohort Studies/
29. Longitudinal Studies/
30. (cohort$ or longitudinal).tw.
31. Cross-Sectional Studies/
32. "Surveys and Questionnaires"/
33. Health Surveys/
34. (survey or surveys).tw.
35. or/28-34
36. 21 and 27 and 35
37. (neonate$ or preterm$ or prematurity or infant$ or child$).tw.
38. visual analog$ scale.tw.
39. (case adj2 report$).tw.
40. (animal or mouse or mice or rat or rats).ti.
41. or/37-40
42. 36 not 41

For peer review only

BMJ Open

The Association Between Vision Impairment and Mortality: Protocol for a Systematic Review and Meta-Analysis

Journal:	*BMJ Open*
Manuscript ID	bmjopen-2020-037556.R1
Article Type:	Protocol
Date Submitted by the Author:	30-Apr-2020
Complete List of Authors:	Ehrlich, Joshua; University of Michigan, Ophthalmology and Visual Sciences; University of Michigan, Institute for Healthcare Policy and Innovation<br>Ramke, Jacqueline; The University of Auckland, School of Optometry and Vision Science; London School of Hygiene and Tropical Medicine, International Centre for Eye Health<br>Macleod, David; London School of Hygiene and Tropical Medicine, International Centre for Eye Health; London School of Hygiene and Tropical Medicine, MRC Tropical Epidemiology Group<br>Swenor, B; Johns Hopkins University, Ophthalmology; Johns Hopkins, Epidemiology<br>Burn, Helen; Stoke Mandeville Hospital<br>Lee, Chan; London School of Hygiene and Tropical Medicine, International Centre for Eye Health; Royal Liverpool University Hospital, St. Paul's Eye Unit<br>Waldock, William; University of Cambridge School of Clinical Medicine<br>Zhang, Justine; London School of Hygiene and Tropical Medicine, International Centre for Eye Health; Manchester Royal Eye Hospital<br>Gordon, Iris; London School of Hygiene and Tropical Medicine, International Centre for Eye Health<br>Congdon, Nathan; Queen's University Belfast; Sun Yat-Sen University Zhongshan Ophthalmic Center<br>Burton, Matthew J; London School of Hygiene and Tropical Medicine, International Centre for Eye Health; Moorfields Eye Hospital<br>Evans, Jennifer; London School of Hygiene and Tropical Medicine, International Centre for Eye Health
**Primary Subject Heading**:	Ophthalmology
Secondary Subject Heading:	Epidemiology, Global health, Public health
Keywords:	OPHTHALMOLOGY, EPIDEMIOLOGY, PUBLIC HEALTH

SCHOLARONE™
Manuscripts

I, the Submitting Author has the right to grant and does grant on behalf of all authors of the Work (as defined in the below author licence), an exclusive licence and/or a non-exclusive licence for contributions from authors who are: i) UK Crown employees; ii) where BMJ has agreed a CC-BY licence shall apply, and/or iii) in accordance with the terms applicable for US Federal Government officers or employees acting as part of their official duties; on a worldwide, perpetual, irrevocable, royalty-free basis to BMJ Publishing Group Ltd ("BMJ") its licensees and where the relevant Journal is co-owned by BMJ to the co-owners of the Journal, to publish the Work in this journal and any other BMJ products and to exploit all rights, as set out in our licence.

The Submitting Author accepts and understands that any supply made under these terms is made by BMJ to the Submitting Author unless you are acting as an employee on behalf of your employer or a postgraduate student of an affiliated institution which is paying any applicable article publishing charge ("APC") for Open Access articles. Where the Submitting Author wishes to make the Work available on an Open Access basis (and intends to pay the relevant APC), the terms of reuse of such Open Access shall be governed by a Creative Commons licence – details of these licences and which Creative Commons licence will apply to this Work are set out in our licence referred to above.

Other than as permitted in any relevant BMJ Author's Self Archiving Policies, I confirm this Work has not been accepted for publication elsewhere, is not being considered for publication elsewhere and does not duplicate material already published. I confirm all authors consent to publication of this Work and authorise the granting of this licence.

For peer review only

The Association Between Vision Impairment and Mortality: Protocol for a Systematic Review and Meta-Analysis

Authors:

Joshua R. Ehrlich[1,2], Jacqueline Ramke[3,4], David Macleod[3,5], Bonnielin K. Swenor[6,7], Helen Burn[8], Chan Ning Lee[3,9], William Waldock[10], Justine H. Zhang[3,11], Iris Gordon[3], Nathan Congdon[12,13,14], Matthew Burton[3,15], Jennifer R. Evans[3]

Affiliations:

1. Department of Ophthalmology and Visual Sciences, University of Michigan, Ann Arbor, MI, USA
2. Institute for Healthcare Policy and Innovation, University of Michigan, Ann Arbor, MI, USA
3. International Centre for Eye Health, London School of Hygiene and Tropical Medicine, London, UK
4. School of Optometry and Vision Science, University of Auckland, Auckland, New Zealand
5. MRC Tropical Epidemiology Group, London School of Hygiene and Tropical Medicine, London, UK
6. Wilmer Eye Institute, Johns Hopkins University School of Medicine, Baltimore, MD, USA
7. Department of Epidemiology, Johns Hopkins Bloomberg School of Public Health, Baltimore, MD, USA
8. Stoke Mandeville Hospital, Aylesbury, UK
9. St Paul's Eye Unit, Royal Liverpool University Hospital, Liverpool, UK
10. University of Cambridge School of Medicine, Cambridge, UK
11. Manchester Royal Eye Hospital, Manchester, UK
12. Queen's University Belfast, Belfast, UK
13. Orbis International, New York, NY, USA
14. Zhongshan Ophthalmic Center, Guangzhou China
15. Moorfields Eye Hospital, London, UK

Corresponding Author: Joshua R. Ehrlich, MD, MPH, 1000 Wall Street, Ann Arbor, MI, 48103, USA. joshre@med.umich.edu, phone: +1 734-763-3732

Keywords: ophthalmology, eye, global health, visual impairment, mortality

For peer review only - http://bmjopen.bmj.com/site/about/guidelines.xhtml

ABSTRACT

Introduction: Due to growth and aging of the world's population, the number of individuals worldwide with vision impairment and blindness is projected to increase rapidly over the coming decades. Vision impairment (VI) and blindness are an important cause of years lived with disability. However, the association of VI and blindness with mortality, including the risk of bias in published studies and certainty of the evidence, has not been adequately studied in an up-to-date systematic review and meta-analysis.

Methods and Analysis: The planned systematic review and meta-analysis will adhere to the Preferred Reporting Items for Systematic Review and Meta-Analysis (PRSIMA) guidelines. Databases, including MEDLINE Ovid, Embase Ovid, and Global Health, will be searched for relevant studies. Two reviewers will then screen studies and review full texts to identify studies for inclusion. Data extraction will be performed, and for included studies the risk of bias and certainty of the evidence will be assessed using the Grades of Recommendation, Assessment, Development, and Evaluation (GRADE) approach. The prognostic factor in this study is visual function, which must have been measured using a standard objective ophthalmic clinical or research instrument. We will use standard criteria from the World Health Organization to categorise VI and blindness. All-cause mortality may be assessed by any method one or more years after baseline assessment of vision. Results from included studies will be meta-analysed according to relevant sections of the Meta-analysis Of Observational Studies in Epidemiology (MOOSE) checklist.

Ethics and Dissemination: This review will only include published data; therefore, ethics approval will not be sought. The findings of this review and meta-analysis will be published in an open-access, peer-reviewed journal and will be included in the ongoing *Lancet Global Health* Commission on Global Eye Health.

Registration details: http://osf.io/weu96

For peer review only

STRENGTHS AND LIMITATIONS OF THIS STUDY

- This is an up-to-date systematic review and meta-analysis to determine the nature and extent of published literature on the association of vision impairment and blindness with mortality.
- This review will comprehensively assess published peer-reviewed English-language manuscripts, with no time period or geographical restrictions.
- This will be the first review to carry out a formal assessment of risk of bias in included studies and the certainty of the evidence on this topic using the Grades of Recommendation, Assessment, Development, and Evaluation (GRADE) approach.
- A potential limitation might be the paucity of published literature on how specific levels of vision impairment contribute to mortality.
- Another potential limitation is that the complexity of pathways between eye health and mortality is unlikely to be fully described and tested in the current literature.

For peer review only

INTRODUCTION

More than 250 million people globally are blind or visually impaired, and the number affected is projected to increase due to growth and aging of the world's population.[1] Poor vision is associated with an increased risk of dementia, depression, falls, and loss of independence.[1,2] Some prior studies have also reported that individuals with vision impairment (VI) have an increased risk of mortality compared to those with normal vision.[3] However, an up-to-date systematic review and meta-analysis of the published literature, including a formal assessment of risk of bias and certainty of the evidence, is needed to characterize the relationship between VI and mortality globally.

In order to guide a systematic review and meta-analysis of the association of VI with mortality, we developed a theoretical framework adapted from the World Health Organization (WHO) International Classification of Functioning (ICF).[4] Our framework illustrates the possible relationship between VI and mortality, as well as the diverse mediating and moderating factors that may contribute to this association (Figure 1). As depicted, we hypothesize that VI, operationalised as a decline in visual function, is associated with mortality through its effects on systemic health (e.g. an increased risk of chronic disease, frailty, and decreased functional status). Factors such as participation (social, physical, daily activities) both impact and are impacted by visual and systemic health (e.g. VI increases the risk of social isolation, which in turn affects overall health). Finally, individual-level traits, environmental and health system characteristics, smoking, and conditions with both ocular and systemic manifestations (e.g. cardiovascular disease, diabetes, hypertension, stroke) may simultaneously increase the risk of both VI and mortality.

A prior systematic review and meta-analysis published in 2016 summarised findings from 29 prospective studies that assessed the association between vision and the risk of mortality.[3] That study reported that the risk of death was 36% higher in the group with the highest level of VI compared to those without VI, and that for each 0.1 increment change in logarithm of minimum angle of resolution (logMAR) the risk of death increased by 4%. However, the study had several limitations. First, it did not assess or account for the level of bias in included studies or certainty of the evidence. Additionally, three included studies assessed VI based on billing codes, and seven used self-reported VI rather objective quantifiable measures. Self-reported visual function may reflect a distinct latent construct,[5] in which case the pooled analysis of studies that assessed visual function objectively and subjectively may bias results in an unpredictable fashion. The highest level of VI was used as a predictor of mortality even though VI categories varied from study to study. Search terms also did not include specific eye conditions (e.g. glaucoma or cataract), so studies of the association between mortality and VI due to these conditions may have been omitted. Finally, several prospective studies have been published in recent years that report the association between VI and mortality in geographic regions, such as sub-Saharan Africa, that were under-represented in the prior systematic review and meta-analysis.

The study described in this protocol will seek to provide an updated review of the literature and estimate of the effect of VI on the risk of mortality. Notably, the complex pathways that may mediate the association between VI and

For peer review only - http://bmjopen.bmj.com/site/about/guidelines.xhtml

mortality may not have been fully described or tested in prior studies, though doing so will be an important future step toward optimizing outcomes for those with VI and blindness. By including an assessment of the risk of bias to inform an overall judgement of the certainty of the evidence, and by considering newly published studies from under-represented geographic regions, this systematic review and meta-analysis will make an important contribution to global eye health.

Objectives / Review questions

This systematic review and meta-analysis will aim to answer the following questions:

1. What is the extent, strength, and quality of the published evidence that VI is associated with the risk of all-cause mortality?
2. To what degree does VI affect the risk of all-cause mortality, and does this risk vary based on level of visual function?

METHODS AND ANALYSIS

Protocol and registration

We have drawn upon the PROGnosis RESearch Strategy (PROGRESS) framework for prognosis research in developing this protocol.[6] The protocol has been registered prospectively with the Open Science Framework (OSF) registry and can be viewed at: http://osf.io/weu96. Any future amendments to the protocol will be noted in the OSF registration. Results of the systematic review and meta-analysis described herein will be reported according to the relevant section of the Preferred Reporting Items for Systematic Review and Meta-Analysis (PRISMA) checklist for systematic reviews.[7] The completed Preferred Reporting Items for Systematic Review and Meta-Analysis Protocols (PRISMA-P) checklist is presented in Appendix 1.[8]

Search method for identification of studies

Electronic searches: We will search the following electronic databases:

1. MEDLINE Ovid (1946 to 2019);
2. Embase Ovid (1980 to 2019); and
3. Global Health (1973 to 2019).

The full electronic search strategy for MEDLINE Ovid is included in Appendix 2. We will not limit the search by date. As noted above, the search will be limited to English language articles and will not include conference abstracts or grey literature.

Searching other resources: We will identify additional studies by searching the reference lists of relevant publications identified through the electronic searches and by searching any prior review articles on this topic.

For peer review only

Criteria for considering studies to review

Types of studies: We will include published prospective and retrospective cohort studies with a baseline assessment of the exposure (vision) and longitudinal assessment of the outcome (all-cause mortality) over a period of at least 1 year. Since age is a strong risk factor for mortality and VI, estimates of the effect of VI on mortality risk must be age-adjusted. Interventional studies and studies where all participants had a specific systemic disease (e.g. diabetes) will be excluded due to the difficulty of separating the possible effect of VI on mortality from the effect of an intervention or systemic disease on mortality. Only peer-reviewed articles published in English will be included. We will not include grey literature or conference abstracts. We will consider publications from all years and geographical regions.

Types of participants*:* Men and women aged 40 years and above at the time of enrolment will be eligible for inclusion. We are restricting the population to this age-group of because of the low rate of mortality in younger individuals.

Types of prognostic factors:

The prognostic factor in this study is visual function. Visual function must have been measured using a standard objective ophthalmic clinical or research instrument, including, but not limited to visual acuity, visual fields, contrast sensitivity, and stereoacuity. If a study contains data on the effect of multiple measures of visual function (e.g. visual acuity and visual fields), we will report each of these and they will be included in the meta-analysis. Studies where visual function was self-reported or determined based on billing codes will not be included.

We will consider several cut-points:

1. For visual acuity, we will consider presenting binocular visual acuity or visual acuity in the better-seeing eye (if both are reported we will use the binocular measurement). Definitions of VI will be based on the categories of VI in the WHO International Classification of Diseases.[9] People with each of the following categories of VI will be compared to those with better vision.
    a. Mild VI or worse will be defined as visual acuity < 6/12.
    b. Moderate VI or worse will be defined as visual acuity <6/18.
    c. Severe VI or worse will be defined as visual acuity <6/60.
    d. Blindness will be defined as visual acuity <3/60.
2. For other measures of vision, we will adopt study-specific definitions of VI since standardised definitions do not exist or are not widely used.

We will use the definitions of VI and blindness reported in the included studies that most closely correspond to these definitions. However, we anticipate heterogeneity in these measures across studies. For example, studies may vary in whether they consider uncorrected, presenting, or best-corrected visual acuity; they may include measures of visual fields, stereoacuity, and/or contrast sensitivity in their definition; they may consider the worse-seeing eye, better-

For peer review only

seeing eye, or binocular visual acuity; and they may employ different categorical definitions of VI and blindness. We will be inclusive but will explore this heterogeneity in meta-regression analyses (see below). When available, we will also use continuous measures of vision (e.g. logMAR or log contrast sensitivity) in our analyses.

Types of outcome measures:

Outcome

1. The outcome is all-cause mortality one or more years after baseline assessment of vision. Mortality may be reported using different measures of effect size and we will include all measures.

Ascertainment of death may be made by any method, including but not limited to review of vital records (e.g. death certificates) or report by an informant. We have chosen to include studies that ascertained death by informant report since not all countries provide access to complete vital records and we seek to include studies from all regions of the world. If sufficient data are available, we will consider performing analyses to determine the association between VI and cause-specific mortality.

Measures of effect

To determine the association between VI and mortality, we will extract age-adjusted measures of effect reported in each included study. All measures of effect must be age-adjusted. When available, we will extract measures that have adjusted for other theoretical confounders (e.g. socioeconomic status, smoking status, cardiovascular disease, diabetes, hypertension, stroke) as depicted in our conceptual framework (Figure). To the extent that it is possible to do so, we will choose measures of effect that are not adjusted for likely mediators on the pathway between VI and mortality, such as overall health or functional status.

Data collection and analysis

Selection of studies: Two review authors will independently screen search results based on title and abstract and will remove reports that clearly do not fall into the scope of this review. Disagreements will be resolved by discussion and consultation with another author as needed. We will acquire the full text of all publications appearing to potentially meet criteria for inclusion in this review. Two review authors will screen all of these reports for method of visual function assessment, type of study design, duration of follow-up, and ascertainment of death. Any disagreements will be discussed and if they cannot be resolved will be arbitrated by a third review author. Screening of search results will be conducted using Covidence systematic review software (Veritas Health Innovation, Melbourne, Australia; available at www.covidence.org).

Data extraction and management: Data extraction will be guided by the relevant sections of the CHecklist for critical Appraisal and data extraction for Systematic Reviews of prediction Modelling Studies (CHARMS) checklist.[10] Two review authors will independently extract the following data from each included study: study design, participant

For peer review only

characteristics, study population and size, study setting, study dates, follow-up duration, diagnostic and ascertainment methods, study attrition, estimates of effect size, and standard errors. Any disagreements will be discussed and if they cannot be resolved will be arbitrated by a third review author. The types of data likely to be reported as estimates of effect size include hazard ratios, risk ratios, rate ratios, standardized mortality ratios, cumulative incidence rates, proportions, survival curves, and/or odds ratios. Data extraction and management will be conducted using Covidence systematic review software.

Assessment of risk of bias: Two reviewers will independently assess the risk of bias in each included study using the Quality in Prognostic Studies (QUIPS) tool.[11] They will assess study participation, attrition, prognostic factor measurement, outcome measurement, confounding, and statistical analysis and reporting. Likely confounders include systemic health conditions that increase risk of VI and mortality (e.g. diabetes), access to medical care, socioeconomic status, and smoking status. For each QUIPS domain, we will assign a rating of low, moderate, or high risk of bias. Ratings of each of the domains in QUIPS will be considered to provide an overall risk of bias assessment for each study. Only studies receiving a rating of low risk of bias in all of the aforementioned domains will be given an overall "low" rating; any study that received one or more ratings of high risk of bias will receive an overall "high" rating; other studies will receive an overall "medium" risk of bias rating.

Measures of association: We will extract summary measures of the association between VI and risk of mortality. We anticipate that some studies will report an overall event rate for the study period, while others may provide estimates of effect size. For all estimates we will extract standard errors; If they are not reported we will extract 95% confidence intervals and use these to calculate standard errors. As noted, we will preference measures that are adjusted for theoretical confounders but not mediators of the association between VI and mortality. We will extract definitions of VI and blindness to permit analyses based on specific levels of VI or blindness, insofar as there are sufficient data available to do so.

Dealing with missing data: We will include studies that follow individuals with and without VI (or with varying levels of VI) for one or more years and report the proportion who died, even if there are missing data. If all of the necessary information are not found in a published study, for articles published in 2010 or later we will email the corresponding author to solicit further information. If we are unable to obtain the necessary information, we will document in the review that we attempted to contact the study authors. We will consider the sensitivity of our meta-analysis to the effect of missing data. We will analyse the data that is available rather than imputing missing data. We will document and discuss the possible effect of missing data on each study and on the overall review and meta-analysis.

Assessment of heterogeneity: Clinical heterogeneity will be assessed by comparing key participant characteristics at the study level (e.g. age, sex, ocular diagnoses). Methodological heterogeneity will also be considered, including a comparison of the risk of bias of included studies. We will assess statistical heterogeneity by inspecting forest plots and through inspection of the I^2 and Tau^2 statistics to examine the proportion of heterogeneity across studies that is

For peer review only - http://bmjopen.bmj.com/site/about/guidelines.xhtml

due to chance. If high levels of heterogeneity are detected (I^2>50%), we will explore likely sources of this heterogeneity (see Meta-regression below). We will also assess small study effects, one of which may be publication bias, by preparing a funnel plot [12], which is a scatter plot of effect size versus precision (standard error).

Data synthesis

Data synthesis and meta-analysis approaches: Methods and results of our meta-analysis will be guided by relevant sections of the Meta-analysis Of Observational Studies in Epidemiology (MOOSE) checklist.[13] Meta-analyses will be performed using a random-effects, generic inverse variance meta-analysis model in Stata version 16 (StataCorp, College Station, TX, USA). Random effects, rather than fixed effects, models will be used since it is likely that the true effect of VI on mortality varies from study to study due to differences in study populations and contexts. The meta-analysis will be summarised using the pooled estimate, its 95% confidence interval, and between study variance (Tau^2). The meta-analysis will be performed and results will be reported for adjusted effect estimates. We will conduct meta-analyses separately for the different types of effect measures (e.g. hazard ratios, odds ratios, and risk ratios). The log of each study estimate and its confidence intervals will be used to determine the study standard error; these will be then pooled using random-effects meta-analysis before taking the exponent of the results to present the pooled effect estimate on the original scale. We will assess and report the overall quality of evidence from our meta-analysis using the modified Grades of Recommendation, Assessment, Development, and Evaluation (GRADE) tool.[14]

Meta-regression: Where data permit, we will investigate the relationship between the following covariates and effect size using random-effects meta-regression:

- Sex
- average age
- method(s) used to measure visual function (visual acuity, visual field)
- duration of follow-up
- global super-region as defined in the Global Burden of Disease study [15]

The meta-regression outcome variable will be the log of the effect estimate for each study, and the aforementioned covariates will be included where data are available to do so.

Sensitivity analyses: We will conduct a sensitivity analysis in which studies are excluded if they are judged to be at high risk of bias.

Patient and Public Involvement Statement

As we plan to review existing published literature only, this review will be performed without specific patient or public involvement.

ETHICS AND DISSEMINATION

For peer review only - http://bmjopen.bmj.com/site/about/guidelines.xhtml

Ethics approval is not required, as our review will only include published data. Findings will be published in an open-access peer-reviewed journal and a summary of results will also be included in the ongoing *Lancet Global Health* Commission on Global Eye Health.[16] We anticipate that the findings will be of considerable interest to those involved in eye health provision, as well as the general medical, public health, development, and governmental sectors.

For peer review only

REFERENCES

1. *World Report on Vision*. Geneva: : World Health Organization 2019. CC BY-NC-SA 3.0 IGO

2. National Academies of Sciences, Engineering, and Medicine. *Making Eye Health a Population Health Imperative: Vision for Tomorrow*. Washington D.C.: : The National Academies Press 2016.

3. Zhang T, Jiang W, Song X, *et al.* The association between visual impairment and the risk of mortality: a meta-analysis of prospective studies. *J Epidemiol Community Health* 2016;**70**:836–42. doi:10.1136/jech-2016-207331

4. World Health Organization. *International Classification of Functioning, Disability and Health*. 1st ed. Geneva, Switzerland: : World Health Organization 2001.

5. Donoghue OA, Ryan H, Duggan E, *et al.* Relationship between fear of falling and mobility varies with visual function among older adults. *Geriatr Gerontol Int* 2014;**14**:827–36. doi:10.1111/ggi.12174

6. Hemingway H, Croft P, Perel P, *et al.* Prognosis research strategy (PROGRESS) 1: A framework for researching clinical outcomes. *BMJ* 2013;**346**. doi:10.1136/bmj.e5595

7. Moher D, Liberati A, Tetzlaff J, *et al.* Preferred reporting items for systematic reviews and meta-analyses: the PRISMA statement. *PLoS Med* 2009;**6**:e1000097. doi:10.1371/journal.pmed.1000097

8. Shamseer L, Moher D, Clarke M, *et al.* Preferred reporting items for systematic review and meta-analysis protocols (PRISMA-P) 2015: elaboration and explanation. *BMJ* 2015;**349**. doi:10.1136/bmj.g7647

9. World Health Organization. *International Classification of Diseases*. 11th rev., ICD-11. Geneva: : World Health Organization 2018.

10. Moons KGM, Groot JAH de, Bouwmeester W, *et al.* Critical Appraisal and Data Extraction for Systematic Reviews of Prediction Modelling Studies: The CHARMS Checklist. *PLOS Medicine* 2014;**11**:e1001744. doi:10.1371/journal.pmed.1001744

11. Hayden JA, van der Windt DA, Cartwright JL, *et al.* Assessing bias in studies of prognostic factors. *Ann Intern Med* 2013;**158**:280–6. doi:10.7326/0003-4819-158-4-201302190-00009

12. Egger M, Davey Smith G, Schneider M, *et al.* Bias in meta-analysis detected by a simple, graphical test. *BMJ* 1997;**315**:629–34. doi:10.1136/bmj.315.7109.629

13. Stroup DF, Berlin JA, Morton SC, *et al.* Meta-analysis of observational studies in epidemiology: a proposal for reporting. Meta-analysis Of Observational Studies in Epidemiology (MOOSE) group. *JAMA* 2000;**283**:2008–12. doi:10.1001/jama.283.15.2008

14. Iorio A, Spencer FA, Falavigna M, *et al.* Use of GRADE for assessment of evidence about prognosis: rating confidence in estimates of event rates in broad categories of patients. *BMJ* 2015;**350**:h870. doi:10.1136/bmj.h870

15. GBD 2017 DALYs and HALE Collaborators. Global, regional, and national disability-adjusted life-years (DALYs) for 359 diseases and injuries and healthy life expectancy (HALE) for 195 countries and territories, 1990-2017: a systematic analysis for the Global Burden of Disease Study 2017. *Lancet* 2018;**392**:1859–922. doi:10.1016/S0140-6736(18)32335-3

16. Burton MJ, Faal HB, Ramke J, *et al.* Announcing The Lancet Global Health Commission on Global Eye Health. *Lancet Glob Health* 2019;**7**:e1612–3. doi:10.1016/S2214-109X(19)30450-4

For peer review only

FIGURE LEGEND

Figure 1. Theoretical framework for the association of vision impairment and mortality. This figure presents the hypothesized relationships between vision impairment and mortality that inform the systematic review and meta-analysis.

For peer review only

Authors' Contributions: All authors made substantive intellectual contributions to the development of this protocol. JRE, JR, MB, and JE conceptualised the review approach. JRE drafted the first version. JR, MB, and JE provided guidance to the research team. DM, BS, IG, NC, HB, CNL, WW, and JZ developed the draft further. All authors were involved in revisions of the manuscript, developing review questions, and the review design. All authors approved the final version of the manuscript. JRE and JE take overall responsibility for the content of this manuscript.

Funding Statement: MJB is supported by grants from the Wellcome Trust (207472/Z/17/Z) to MB. JR is a Commonwealth Rutherford Fellow, funded by the UK government through the Commonwealth Scholarship Commission in the UK. BKS is supported by the National Institutes of Health (K01AG052640). JRE is supported by the National Institutes of Health (K23EY027848). The Lancet Global Health Commission on Global Eye Health is supported by The Queen Elizabeth Diamond Jubilee Trust, Moorfields Eye Charity (GR001061), NIHR Moorfields Biomedical Research Centre, The Wellcome Trust, Sightsavers, The Fred Hollows Foundation, The SEVA Foundation, The British Council for the Prevention of Blindness and Christian Blind Mission. No funder had any role in the design or conduct of this work.

Acknowledgements: None.

Competing Interests: None declared.

Word Count: 2,957

For peer review only

For peer review only - http://bmjopen.bmj.com/site/about/guidelines.xhtml

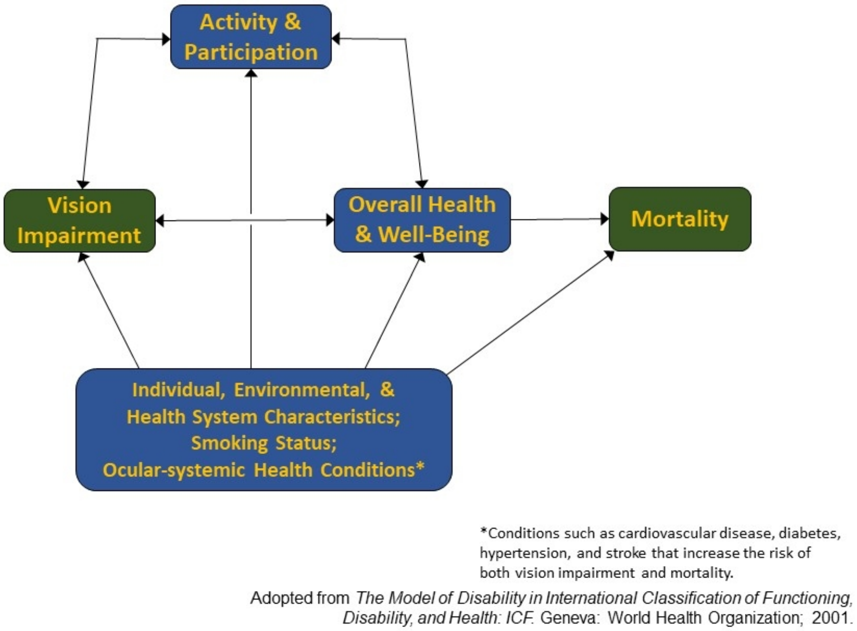

Theoretical framework for the association of vision impairment and mortality. This figure presents the hypothesized relationships between vision impairment and mortality that inform the systematic review and meta-analysis.

254x190mm (96 x 96 DPI)

Appendix 1. PRISMA-P 2015 Checklist

Section/topic	#	Checklist item	Information reported: Yes	Information reported: No	Line number(s)
**ADMINISTRATIVE INFORMATION**					
**Title**					
Identification	1a	Identify the report as a protocol of a systematic review	☒	☐	1, 27, 47, 65, 99
Update	1b	If the protocol is for an update of a previous systematic review, identify as such	☒	☐	94
**Registration**	2	If registered, provide the name of the registry (e.g., PROSPERO) and registration number in the Abstract	☒	☐	36, 112
**Authors**					
Contact	3a	Provide name, institutional affiliation, and e-mail address of all protocol authors; provide physical mailing address of corresponding author	☒	☐	5-14
Contributions	3b	Describe contributions of protocol authors and identify the guarantor of the review	☒	☐	324-328
**Amendments**	4	If the protocol represents an amendment of a previously completed or published protocol, identify as such and list changes; otherwise, state plan for documenting important protocol amendments	☐	☒	N/A
**Support**					
Sources	5a	Indicate sources of financial or other support for the review	☒	☐	330-337
Sponsor	5b	Provide name for the review funder and/or sponsor	☒	☐	330-337
Role of sponsor/funder	5c	Describe roles of funder(s), sponsor(s), and/or institution(s), if any, in developing the protocol	☒	☐	336-337
**INTRODUCTION**					
**Rationale**	6	Describe the rationale for the review in the context of what is already known	☒	☐	94-97
**Objectives**	7	Provide an explicit statement of the question(s) the review will address with reference to participants, interventions, comparators, and outcomes (PICO)	☒	☐	103-107
**METHODS**					
**Eligibility criteria**	8	Specify the study characteristics (e.g., PICO, study design, setting, time frame) and report characteristics (e.g., years considered, language, publication status) to be used as criteria for eligibility for the review	☒	☐	132-149
**Information sources**	9	Describe all intended information sources (e.g., electronic databases, contact with study authors, trial registers, or other grey literature sources) with planned dates of coverage	☒	☐	121-130
**Search strategy**	10	Present draft of search strategy to be used for at least one electronic database, including planned limits, such that it could be repeated	☒	☐	Appendix 2

For peer review only

Section/topic	#	Checklist item	Information reported Yes	Information reported No	Line number(s)
STUDY RECORDS					
Data management	11a	Describe the mechanism(s) that will be used to manage records and data throughout the review	☒	☐	200-208
Selection process	11b	State the process that will be used for selecting studies (e.g., two independent reviewers) through each phase of the review (i.e., screening, eligibility, and inclusion in meta-analysis)	☒	☐	191-198
Data collection process	11c	Describe planned method of extracting data from reports (e.g., piloting forms, done independently, in duplicate), any processes for obtaining and confirming data from investigators	☒	☐	200-208
**Data items**	12	List and define all variables for which data will be sought (e.g., PICO items, funding sources), any pre-planned data assumptions and simplifications	☒	☐	205-207
**Outcomes and prioritization**	13	List and define all outcomes for which data will be sought, including prioritization of main and additional outcomes, with rationale	☒	☐	163-169
**Risk of bias in individual studies**	14	Describe anticipated methods for assessing risk of bias of individual studies, including whether this will be done at the outcome or study level, or both; state how this information will be used in data synthesis	☒	☐	210-218
DATA					
**Synthesis**	15a	Describe criteria under which study data will be quantitatively synthesized	☒	☐	245-256
	15b	If data are appropriate for quantitative synthesis, describe planned summary measures, methods of handling data, and methods of combining data from studies, including any planned exploration of consistency (e.g., I^2, Kendall's tau)	☒	☐	220-226, 236-242, 245-256
	15c	Describe any proposed additional analyses (e.g., sensitivity or subgroup analyses, meta-regression)	☒	☐	258-266
	15d	If quantitative synthesis is not appropriate, describe the type of summary planned	☐	☒	N/A
**Meta-bias(es)**	16	Specify any planned assessment of meta-bias(es) (e.g., publication bias across studies, selective reporting within studies)	☒	☐	241-242
**Confidence in cumulative evidence**	17	Describe how the strength of the body of evidence will be assessed (e.g., GRADE)	☒	☐	245-246

For peer review only

Appendix 2: MEDLINE Ovid search strategy

1. exp eye diseases/
2. Visually Impaired Persons/
3. ((low$ or handicap$ or subnormal$ or impair$ or partial$ or disab$ or disorder$ or loss$ or limit$) adj3 (vision or visual$ or sight$)).tw.
4. ((macul$ or retina$ or choroid$) adj3 degener$).tw.
5. ((macul$ or retina$ or choroid$) adj3 neovasc$).tw.
6. (AMD or ARMD).tw.
7. ((diabet$ or proliferative or non-proliferative or pre-proliferative) adj4 retinopath$).tw.
8. (diabet$ adj2 (eye$ or vision or visual$ or sight$)).tw.
9. glaucoma$.tw.
10. cataract$.tw.
11. blindness.tw.
12. Visual Acuity/
13. visual acuit$.tw.
14. Contrast Sensitivity/
15. (contrast adj2 sensitivity).tw.
16. Depth Perception/
17. stereopsis.tw.
18. (stereo adj1 acuit$).tw.
19. Visual Fields/
20. ((visual$ or vision) adj2 function$).tw.
21. or/1-20
22. exp Mortality/
23. Death Certificates/
24. mortality.tw.
25. death$.tw.
26. (fatality or fatalities).tw.
27. or/22-26
28. Cohort Studies/
29. Longitudinal Studies/
30. (cohort$ or longitudinal).tw.
31. Cross-Sectional Studies/
32. "Surveys and Questionnaires"/
33. Health Surveys/
34. (survey or surveys).tw.
35. or/28-34
36. 21 and 27 and 35
37. (neonate$ or preterm$ or prematurity or infant$ or child$).tw.
38. visual analog$ scale.tw.
39. (case adj2 report$).tw.
40. (animal or mouse or mice or rat or rats).ti.
41. or/37-40
42. 36 not 41

For peer review only
